# Health care providers’ preparedness and health care protection against the third wave of COVID-19 pandemics in a resource-limited setting in Southwest Ethiopia: a multi-center cross-sectional study

**DOI:** 10.11604/pamj.2023.44.53.31428

**Published:** 2023-01-26

**Authors:** Fisha Alebel GebreEyesus, Omega Tolessa Geleta, Bisrat Zeleke Shiferaw, Tadesse Tsehay Tarekegn, Baye Tsegaye Amlak, Mamo Solomon Emeria, Tamene Fetene Terefe, Bogale Chekole Temere, Agerie Aynalem Mewahegn, Melkamu Senbeta Jimma, Ermias Sisay Chanie, Natnael Moges Misganaw, Fatuma Seid Degu, Menen Amare Eshetu

**Affiliations:** 1 Department of Nursing, College of Medicine and Health Sciences, Wolkite University, Wolkite, Ethiopia,; 2 Department of Nursing, College of Health Sciences, Debre Markos University, Debre Markos, Ethiopia,; 3 Department of Pediatrics and Child Health Nursing, College of Medicine and Health Science, Injibara University, Injibara, Ethiopia,; 4 Department of Nursing, College of Health Sciences, Assosa University, Assosa, Ethiopia,; 5 Department of Pediatric and Neonatal Nursing, College of Health Sciences, Debre Tabor University, DebreTabor, Ethiopia,; 6 Department of Nursing, College of Medicine and Health Science, Wollo University, Wollo, Ethiopia,; 7 Department of Nursing, College of Medicine and Health Science, Mizan Tepi University, Mizan Tepi, Ethiopia

**Keywords:** Healthcare providers, health care workers, preparedness, COVID-19, Gurage

## Abstract

**Introduction:**

the provision of quality health care during the COVID-19 pandemic depends largely on the health of health care providers. Health care providers as frontline caregivers dealing with infected patients play a significant role in limiting the outbreak of the disease by implementing safety and prevention practices. However, low and middle-income countries experience barriers to preparedness due to limited resources.

**Methods:**

an institutional-based cross-sectional study was conducted among 326 health care providers from August 10-25, 2021 in Gurage zonal public hospitals. A simple random sampling technique was used to select the study participants. A pretested self -administered structured questionnaire was used as a data collection tool. The data were entered into the Epi-data 3.1 and exported to Statistical package for the social sciences (SPSS) version 25.0 for analysis. Both descriptive statistics and inferential statistics were presented.

**Results:**

this study showed that 53.1%, of health care providers, had adequate preparation against COVID-19 pandemics. The finding showed that monthly income, occupation, and working experience were found to be significantly associated with health care providers’ preparedness. Nearly one-quarter (24.8%), 28.3%, 34.5%, and 39.8% of health care providers had access to facemasks, alcohol sanitizer, glove, and isolation gowns respectively.

**Conclusion:**

the levels of health care providers’ preparedness and health care protection against the third wave COVID-19 pandemic were found to be low. Based on our findings, the government and other stakeholders should design interventions to increase health care providers' preparedness to respond to the ongoing pandemic and purchase an adequate supply of personal protective equipment to protect the health care providers.

## Introduction

On December 31^st^, 2019, the World Health Organization (WHO) contacted China about media reports of a cluster of viral pneumonia in Wuhan, later attributed to a coronavirus, now named SARS-CoV-2 [1]. The outbreak soon spread to the whole country and was declared as a public health emergency of international concern by WHO in late January 2020 [2]. And recognized as a global pandemic on 11^th^ March 2020 [3]. The WHO and other international health organizations often refer to “waves of a pandemic”. A wave refers to a rising number of COVID-19 cases that has a specific peak and decline [4,5]. The third wave of COVID-19 infection was first reported in the UK (United Kingdom) at the end of January 2020 and at least three-quarters of cases were the new variant firstly discovered in India [6]. The third wave occurs when a third peak is observed in a population and often occurs as a result of social determinants of health. This wave often worsens economic and health care inequalities. And put its effects on the next generation [7-9]. Healthcare providers (HCPs) are always at the forefront in the response to emerging infectious disease outbreaks. So, they are at a higher risk of being infected with SARS-CoV-2 than the general population [10]. Likewise, HCPs who become infected may be a source of infection to their friends, family members, and other vulnerable people under their care [11].

COVID-19 has so far affected many health professionals around the world [12,13]. On average, 14% of all cases of COVID-19 worldwide are in healthcare providers. A recent report from the International Council of Nurses (ICN), which surveyed 50 countries, mostly from Europe and the Americas, found that health worker infections ranged from 1-32% of all confirmed COVID-19 cases [14]. Besides this, ICN reported that HCPs are at high risk of infection because of the unavailability of personal protective equipment (PPE), medical supplies, and inadequate preparation for this pandemic [15]. Health care providers as the frontline caregivers dealing with infected patients play a crucial role in limiting the outbreak of the disease by implementing safety and prevention practices set by the WHO and Center for Disease Control and Prevention (CDC) recommendations for infection prevention and epidemic preparedness and response plan [16,17]. Despite its recommendation, low-resource countries, like Ethiopia, experience multilevel barriers to preparedness [18]. It's implementation is also challenged by a lack of public health planning and preparedness, ineffective institutional infection prevention measures, shortage of guidance in infection control rule procedures, shortage of personal protective equipment, lack of isolation facilities, environmental contamination, and lack of awareness on the preventive measures against COVID-19 may also contribute to the increase in COVID-19 cases among HCPs [12,19-22]. Mitigating and reducing this risk is essential to protecting their well-being and reducing the spread of COVID-19 [1].

The provision of quality health care during the COVID-19 pandemic depends largely on the health of health care providers [23]. Available scientific evidence suggests that appropriate PPE use, hand hygiene best practices, implementation of universal masking policies in health care facilities, and adequate infection prevention and control (IPC) training and education are associated with decreased risk of COVID-19 among health workers [1,16,17,24,25]. Despite the extensive efforts made so far to curb the COVID-19 pandemics in Africa, the COVID-19 pandemic is rampantly rising today due to limited resources and weak healthcare system capacity [26-28]. Moreover, coronavirus cases have been rising since the start of the third wave in May. Sixteen African countries are now seeing a resurgence of the virus, with the more contagious delta strain detected in 10 of them [29]. Ethiopia is one of the countries threatened by COVID- 19, with a total of 303,171 confirmed cases and 4,618 registered deaths as of August 27/2021. It is now the leading country in East Africa with the highest number of infected people [30]. Thousands of HCPs have been infected with COVID-19 [31]. This demands more stringent measures to combat the pandemic and reduce mortality in this population [32]. Besides this Ethiopian ministry of health announced that the third wave of coronavirus has reached an alarming stage and spreading across the country on August 24^th^, 2021 [33]. By 2017, in Ethiopia, there is a total of 118,507 healthcare providers working in a different corner of the country with a proportion of 1.26 healthcare providers per 1000 population [34]. Which is by far less than the minimum threshold of 4.45 per 1000 population set by WHO, which is a double burden for the country [35]. As a result, key components of effectively providing primary care healthcare have been challenged while managing the COVID-19 pandemic [36]. Even though the number of COVID-19 cases in Ethiopia is rising rapidly and became a serious threat to the healthcare system, there is a scarcity of information on the health care providers´ preparedness and health care protection against the third wave of COVID-19 pandemics in the country as a whole and the study area in the particular. Therefore, this study aims to assess health care providers´ preparedness and health care protection against the third wave of COVID-19 pandemics in a resource-limited setting in Southwest Ethiopia, in 2021.

**Objectives:** to assess health care providers´ preparedness against the third wave of COVID-19 pandemics; to determine the availability of PPE to protect health care providers against COVID-19 pandemics in a resource-limited setting in Southwest Ethiopia, 2021.

## Methods

**Study design:** an institutional-based cross-sectional study design was conducted.

**Study period and setting:** the study was conducted in the Gurage zonal public health institutions of Southern Nations, Nationalities, and Peoples' Region (SNNPR) from August 10-25/2021. A Gurage zone is one of the fifteen zones and four special woredas found in the SNNPR State. Wolkite town is the capital of the Gurage zone, which is located 158 km southwest of Addis Ababa and 260 km from Hawasa. It has 20 woredas and two municipalities. According to the 2012 population projection by the central statistics agency (CSA) the total population is 1,767,518. There are seven hospitals in the Gurage zone. Five of the hospitals in the zone are primary hospitals, one general hospital and the remaining one is a comprehensive, specialized hospital, there are 79 health centers 7 are NGO HC and 444 Functional health posts serving the total population in the zone. There is also a COVID-19 testing center; some hospitals are readily organized to serve quarantine and treatment with 100 consecrated beds [37].

**Study participants:** the randomly selected health care providers from the selected public health hospitals were the study population and all health care providers who are working in the selected public health hospitals were included while those health care providers who are mentally/critically ill and on annual leave were excluded from the study.

**Sample size:** the minimum sample size was determined by using a single population proportion formula


$$n=\frac{{\left(Z^{a}/2\right)}^{2}p\left(1-p\right)}{d^{2}}$$

by assuming a 95% confidence level (Z a/2 = 1.96), a margin of error of 5%, P=proportion of health care providers who are prepared for the pandemic in Libya (26.3%) [38] and a 10% addition for non-response rate. The final sample size became 326.

**Sampling technique and procedure:** in the study area, there are six public and one non-governmental hospital. From these hospitals, we have included six public hospitals and all health care providers working in each department. We have got the numbers of health care providers working in each hospital from the human resource office of the respective hospitals and use them as a sampling frame. The sample size in each hospital was allocated proportionally to the number of health professionals. The study participants were selected using simple random sampling techniques. Within each hospital, the sample was taken from each department based on the proportion of their health professionals.

### Variables

**Dependent variable:** health care providers’ preparedness yes/no; availability of PPE always available/not always available.

**Independent variable:** age, gender, religion, ethnicity, levels of education, marital status, job category, residence, monthly income, work experience, working setup, presence of infected colleague, presence of infected family members.

**Data collection procedure:** data were collected through a pre-tested, structured, and a self-administered questionnaire designed based on extensive literature review, course material regarding emerging respiratory diseases, and guidelines issued by the Ethiopian federal ministry of health [39] and, the Ethiopian public health institute [17], from different healthcare providers in their respective wards by trained and experienced personnel´s. A minimum of two-meter distance was kept between HCPs and data collectors. In addition to this, the data collectors wear protective face masks. A questionnaire was developed and tested for reliability and validity and accordingly; the cronbach alpha coefficient was found to be 0.703. Besides, a pretest was done before actual data collection on 5% of a similar population in one hospital not included in the actual data collection to assess flow, readability, and clarity of the questionnaire. The questionnaire consisted of three main themes: the first part consisted of the sociodemographic background of the study participants including gender, age, educational status, marital status, job description, state of residence, religion, ethnicity, monthly income, working setup, presence of infected colleague, and presence of infected family members. The second part assesses the availability of personal protective equipment using eight items rated on a five-point Likert scale as [1] never available, [2] rarely available, [3] sometimes available, [4] often available, and [5] always available. For ease of analysis, the Likert scale was summarized as “always available” and “not always available” (those respondents who often replied available, sometimes available, rarely available, and never available) [40].

The third part comprised 11 items to assess the overall health care providers´ preparedness in terms of managing cases of COVID-19 infection. The questionnaire evaluates training experience with COVID-19, diagnosis, and management of COVID-19 patients, use of PPE, safety precautions, isolation procedures, measures to prevent infection, and reporting procedures. Those who scored ≥8 on the preparedness scale had adequate preparedness [38]. Eight data collectors and two supervisors were recruited for data collection, who have experience in data collection. To keep data quality supervisors and data collectors were oriented on how and what information they should collect from the targeted data sources. The completeness and consistency of the collected data were checked daily during data collection by the supervisor and the principal investigator. Whenever there appear incompleteness and ambiguity of recording, the filled information formats were cross-checked with source data soon. Individual records with incomplete data were also excluded.

**Data processing and analysis:** the data were cleaned, coded, and entered into EpiData 3.1 and then exported to SPSS version 25.0 statistical package for further analysis. Data cleaning was performed to check for accuracy, consistencies, and missing values and variables. Descriptive statistics and inferential statistics chi-square tests were carried out to illustrate the percentage and frequencies of study variables. Both bivariable and multivariable analyses were used to see the association of different variables. Those variables which revealed a statistically significant value at a p-value of ≤0.25 in the bivariable analysis were selected for multivariable logistic regression. For model fit, Hosmer and Lemeshow test was carried out and found to be (0.515) which indicated the final model was well-fitted and multi-collinearity was also assessed. An adjusted odds' ratio with a 95% confidence interval was used to measure the degree of association between variables. A p-value of ≤ 0.05 was considered statistically significant during multivariable logistic regression.

**Ethics approval and consent to participate:** ethical clearance approval was obtained from Wolkite University, Ethical Review Committee. Then data were collected after getting an official letter from the zonal health department. The purpose of the study was explained to the study participants; anonymity, privacy, and confidentiality were ensured. Before data collection, informed verbal consent was obtained from the study participants. The respondents´ right to refuse or withdraw from participating in the study was also fully acknowledged.

## Results

**Socio-demographic characteristics of the respondents:** there were 322 study participants involved in the study with a response rate of 98.8%. The highest proportion of respondents 157 (48.8%) was within the age group of 26-30 years with a mean age of 28.71 with a standard deviation (SD) ±5.288. It showed that there was nearly equal participation of males (51.9%) and females (48.1). Around two-thirds of the participants were Gurage (64.6%) followed by Amhara (17.1%) and Oromo (10.2%). Half of the participants (49.7%) were orthodox Christian and 56.5 % of the participants were married. Regarding the educational status of the respondents, 57.8 % (186) were degree holders followed by 34.2% (110) diplomas. Concerning job description, more than one -thirds (35.1%) of the participants were nurses followed by the pharmacy (11.8%) and general practitioner (11.5%). Around two-thirds (66.1%) of the participants had ≤5 years of working experience with a mean experience of 4.7925 SD± 3.58. One-third of the participants (34.5 %) were employed at a general hospital. Nearly half of the participants (47.2%) of health care providers were living with their spouses. Most of the health care providers were practiced at the medical ward and medical outpatient department (13.3%) followed by the emergency ward (11.8%) (Table 1).

**Table 1:** socio-demographic characteristics of frontline healthcare providers in Gurage Zonal public hospitals, Southwest Ethiopia, 2021

Variables	Categories	Number	Percent
Age	18-25	92	28.6
	26-30	157	48.8
	31-40	60	18.6
	>40	13	4.0
Sex	Male	167	51.9
	Female	155	48.1
Marital status	Married	182	56.5
	Single	140	43.5
Educational status	Diploma	110	34.2
	Degree	186	57.8
	Master’s degree and above	26	8.1
Job description	Nurse	113	35.1
	Physicians	44	13.7
	Midwifery	34	10.6
	Pharmacy	38	11.8
	Lab Tech	16	5.0
	HO	34	10.6
	Environmental health	10	3.1
	Others	33	10.2
Average monthly income	<60	32	9.9
	60-100	110	34.2
	100-140	119	37.0
	>140 USD	61	18.9
Year of service (experience)	≤5	213	66.1
	>5	109	33.9
Types of hospital	Primary	85	26.4
	General	111	34.5
	Referral	47	14.6
	Isolation center	79	24.5
Have you, infected colleagues	Yes	96	29.7
	No	226	70.0
Have you, infected family member	Yes	38	11.8
	No	284	87.9

**Figure 1:**
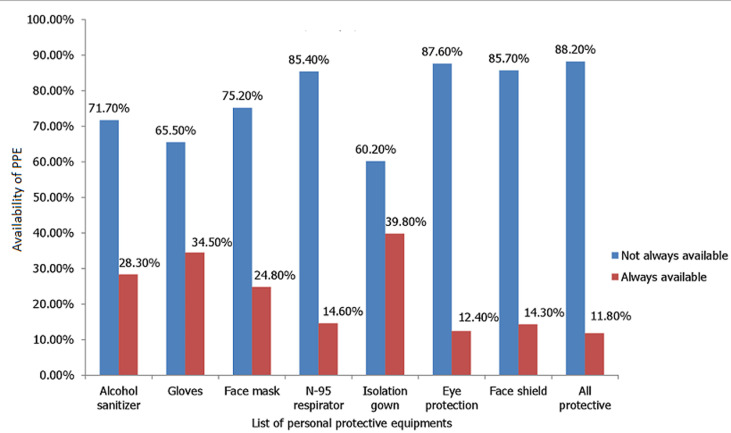
availability of personal protective equipment for health care providers in Gurage Zone public hospitals, SNNPR, South West, Ethiopia, 2021

**Availability of personal protective equipment and Healthcare providers’ preparedness:** in this study, 28.3% (91), more than one-thirds (34.5%), and around two-fifths (39.8 %) of the study participants said that alcohol sanitizers, gloves, and isolation gowns were always available at their health institution respectively. On the other hand, the majority of the study participants replied that eye protection (87.6%), face shields (85.7%), and N95 respirator (85.4%) were not always available at their institution respectively. Only one-fourth (24.8%) of the participants responded that face masks were always available (Figure 1).

**Health care providers preparedness against COVID-19 pandemic:** regarding health care providers' preparedness, three-fifths (60.9%) of HCPs were participating in a training course for outbreak management and more than three-fourths (78.3%) of them were prepared to manage the COVID-19 outbreak. Besides this, 53.1% and 57.5% of HCPs were familiar with isolation procedures and how to report potential COVID-19 cases respectively. Around two-thirds (66.1 %) and three-fourths (73.6 %) of HCPs responded that there was protocol for triage and isolation of suspected cases as well as the availability of isolation rooms respectively. Sixty-six point five percent of HCPs thought that their institutions were prepared for the COVID-19 outbreak. The majority (88.2 %) of the participants were knowledgeable about the safety precautions that should be taken from aerosol transmission in patients with COVID-19 and more than half of HCPs (54.3%) were familiar with the criteria to guide the evaluation of persons under investigation (Table 2). Generally, 171 (53.1 %) of health care providers´ were adequately prepared.

**Table 2:** level of preparedness against COVID-19 pandemics among frontline healthcare providers in Gurage Zonal public hospitals, Southwest Ethiopia, 2021

Variables	Categories	
	Yes (%)	No (%)
Have you participated in a training course for outbreak management?	196 (60.9)	126 (39.1)
Protocol for triage and isolation of suspected cases?	213 (66.1)	109 (33.9)
Availability of isolation room?	237 (73.6)	85 (26.4)
Are you prepared to manage the COVID-19 outbreak?	252 (78.3)	70 (21.7)
Do you consider your hospital to be prepared for the COVID-19 outbreak?	214 (66.5)	108 (33.5)
Are you prepared to properly use PPE?	257 (79.8)	65 (20.2)
Do you know the isolation procedure?	171 (53.1)	151(46.9)
Do you know how to report a potential COVID-19 case?	185 (57.5)	137 (42.5)
Do you know what to do if you have signs of the COVID-19 infection?	271 (84.2)	51 (15.8)
Do you know the safety precautions that should be taken for aerosol transmission in patients with COVID-19?	284(88.2)	38 (11.8)
Do you know the criteria to guide the evaluation of persons under investigation?	175 (54.3)	147 (45.7)

**Factors associated with health care providers preparedness among health care providers in Gurage Zone public hospitals, SNNPR, South West, Ethiopia, 2021:** first bivariable logistic regression analysis was conducted to detect the presence of association and measure the relative effect of each independent variable overall health care providers´ preparedness against COVID-19 pandemics. As a result, among all other variables, age, ethnicity, occupation, types of health facility, monthly income, experience, and presence of infected family were found to have an association (i.e. p-value of ≤ 0.25) and become eligible for multivariable analysis. Then, the multivariable logistic regression analysis showed that monthly income, occupation, and experience were found to be statistically significant predictors of overall health care providers´ preparedness. Health care providers´ whose monthly income 60-100, 100-140, and >140 USD were about 2.72 AOR=2.724; 95% CI (1.107-6.703), 2.80 AOR=2.808; 95% CI (1.142-6.906), and 3.82 AOR=3.823; 95% CI (1.315-11.113) times adequately prepared for COVID-19 pandemic respectively than health care providers´ whose monthly income < 60 USD. Regarding the occupation of health care providers, lab technicians were about 27% AOR=0.268; 95% CI (0.079-0.902) less likely prepared for the COVID-19 pandemic than nurses. Health care providers whose work experience was less than 5 years were about 1.74 AOR=1.735; 95% CI (1.047-2.878) times more likely prepared for a COVID-19 pandemic than those whose work experience greater than 5 years (Table 3).

**Table 3:** factors associated with health care providers preparedness among health care providers In Gurage Zone public hospitals, SNNPR, South West, Ethiopia, 2021

Variable	Over all preparedness		COR (95%, CI)	AOR (95%, CI)
	No adequate preparedness n (%)	Adequate preparedness n (%)		
**Monthly income**				
<60	23 (6.4)	9 (2.8)	1.00	1.00
60-100	54 (16.77)	56 (17.4)	2.650 (1.125-6.241)*	2.724(1.107-6.703)**
100-140	55 (17.08)	64 (19.87)	2.974 (1.270-6.962)*	2.808 (1.142-6.906)**
>140 USD	19 (5.9)	42 (13.04)	5.649 (2.202-14.492)*	3.823 (1.315-11.113)**
**Occupation**				
Nurse	53 (16.46)	60 (18.64)	1.00	1.00
Physician	10 (3.1)	34 (10.56)	3.003 (1.355-6.657)*	2.411 (0.935-6.216)
Midwifery	14 (4.2)	20 (6.2)	1.262 (0.580-2.743)	1.1336 (0.589-3.030)
Pharmacy	21 (6.52)	17 (5.28)	0.715 (0.342-1.497)	0.718 (0.336-1.534)
Lab technician	12 (3.72)	4 (1.24)	0.294 (0.090-0.968)*	0.268 (0.079-0.902)**
Health officer	17 (5.28)	17 (5.28)	0.883 (0.410-1.902)	0.816 (0.358-1.860)
Environmental Health	5 (1.57)	5(1.57)	0.883(0.242-3.220)	1.187 (0.308-4.578)
**Others**	19(5.9)	14 (4.2)	0.651 (0.297-1.424)	0.686 (0.304-1.546)
Experience				
0-5	87 (27.02)	126 (39.13)	2.060 (1.289-3.293)*	1.735 (1.047-2.878)*
>5	64 (19.88)	45(13.98)	1.00	1.00

## Discussion

The results of this study had shown that 53.1%, of health care providers´, had adequate preparation against COVID-19 pandemics. This is higher than the finding reported from the study carried out in Addis Ababa (33.6%) [41], Debre Tabor (41.3%) [42], Libya (20.6%) doctors and (26.3%) nurses [38], Ghana (27.8%) [43], Palestine (11.6%) [41], Saudi Arabia (33%) [44], Pakistan (25%) [45], Jordan (49%) [46] and India (41.8%) [47]. The possible explanation for the disparity might be due to the difference in the data collection period, the difference in data collection tools used to assess preparedness, a difference in educational level and personal characteristics. In line with our study, a study performed in North Showa zone healthcare facilities, Ethiopia [48], and Offinso-North District, Ghana [49] indicated that 53.4% and 57.5 % of health care providers were adequately prepared respectively. Our finding was slightly lower than those of a study done in selected hospitals of Southwest Ethiopia (59.5%) [37], selected health facilities in a resource-limited setting in Addis Ababa, Ethiopia [50], where 58.6% medical laboratory, 61.5% nurses/midwives and 67.2% pharmacist were adequately prepared, Yemen (60%) [51], Lebanon (67%) [52], United Arab Emirates (87.9%) [53] and Australia (75.7%) [54]. The possible justification for the difference could be due to a difference in study participants´ socioeconomic status. Country context and disease burden.

Our finding showed that health care providers who had less than five years of working experience were about two times AOR=1.735; 95% CI (1.047-2.878) more likely to be prepared for a COVID-19 pandemic than those who had more than five years of working experience. This could be because as the health care providers´ experience increases, the exposure, and fear for such outbreaks will reduce, they also tend to be negligent, seek paramount incentives, and positive reinforcement to become well-prepared. Contrary to our finding, another study from, Debre Tabor, Ethiopia [42], United Arab Emirates [53], and Nepal [55] showed that hospital health care providers with more clinical experience were significantly more prepared for the COVID outbreak. This study also investigated that laboratory technicians were about 27% AOR=0.268; 95% CI (0.079-0.902) less likely prepared for the COVID-19 pandemic than nurses. This can also be interpreted as nurses being 3.7 times more prepared to combat COVID-19 pandemics than laboratory technicians. This study was supported in a qualitative study conducted in China [56] which stated that nurses´ professional values are the basis of their working attitude and motivations, which have a positive impact on their work enthusiasm, willingness, and preparedness too. Moreover, nurses took the initiative to the adjustment of self-psychology, respond to moral dilemmas, actively seek knowledge, and actively communicate with patients and their families in a targeted manner, which improved the understanding of the patients and their families regarding COVID-19 and subsequently improved the patients´ compliance. Furthermore, in our study, the majority of nurses have participated in COVID-19 training this will enhance their preparedness to avert COVID-19 infection. This is supported by a study carried out in the United Arab Emirates [53] which revealed that participants who completed infection control training and COVID-19 training were more prepared for the COVID-19 outbreak,

The current study also revealed that health care providers whose monthly income 60-100, 100-140, and >140 USD were about three times AOR=2.724; 95%CI (1.107 6.703), three times AOR=2.808; 95%CI (1.142-6.906) and four times AOR=3.823; 95%CI (1.315-11.113) more likely to be adequately prepared for COVID-19 pandemic respectively than health care providers whose monthly income <60 USD. This implied that as income increases, their preparedness also increases. As we know those health care providers who had completed masters or doctoral studies had the highest income. In line with this, the study conducted in Serbia [57] showed that those respondents who completed masters or doctoral studies expressed the highest rates of self-assessed individual preparedness and it also pointed to the importance of an advanced and continued training education program about infection control and use of appropriate measures which will enhance individual preparedness to tackle the COVID-19 pandemics. Besides this in our study, there is low availability of PPE; as a result of this, those health care providers who had less monthly income can´t afford to buy PPE, which in turn reduces their preparedness. Furthermore, a qualitative study conducted in Oman [58] showed that those individuals who lived in low socioeconomic conditions were more vulnerable to the disease, prone to financial depression and they are ill-prepared to tackle COVID-19 pandemics. As the coronavirus disease 2019 pandemic increased exponentially, the health care system worldwide was challenged with a potential scarcity of personal protective equipment (PPE). Protecting the spread of infection to and from healthcare providers and patients depends on the effective use of PPE: alcohol sanitizer, gloves, facemasks, eye protection, face shields, N-95 respirators, and isolation gowns. A serious shortage of these valuable PPE puts health care providers at high risk of acquiring and transmitting infection and became a major obstacle for adequate preparedness.

In our multi-center institutional-based study the availability of personal protective equipment showed that 24.8%, 28.3%, 34.5%, and 39.8% of health care providers always had access to facemasks, alcohol sanitizers, gloves, and isolation gowns respectively. On the other hand, the majority of HCPs did not find N95 respirator (85.4%), face shields (85.7%), and eye protection (87.6%) when needed respectively. This is nearly similar to a study carried out in North showa [18], where nearly one-third (31%), 27.4%, 15.9%, 14.5%, and 14.2% of HCPs had access to gloves, facemask, goggle, shoe, and apron respectively in hospitals. Unlike our finding, a study from Tanzania [59] found that there was a better availability of medical masks (28.7%), latex gloves (85.7 %), and alcohol-based hand rubs (33.6%). Another study carried out in Pakistan [60] showed there was an adequate supply of disposable gloves (87.6%), isolation gowns (56.6%), and eye-protective equipment (27.2%). Similarly, the study conducted in Jordan [46] showed that face masks (33.8%), goggles (34.1%), isolation gowns (69.5%), and gloves (80.8%) were always available in their institution. Interestingly, a study conducted in Saudi Arabia [44] showed that all participating health care providers reported that they have adequate supplies of personal protective equipment´s (PPEs), such as goggles, masks, and gowns, to manage emergencies, 99.72% of HCWs depend on an external resource center like CDC and WHO for the required emergency materials.

In another study in the Hail region, Saudi Arabia [61] revealed that there was sufficient (68.5%) availability of PPE in their unit. Moreover, our study result was lower than in a study carried out in Palestine [40], where 48.6% and 51.4% of surveyed HCPs indicated that they always had alcohol-based sanitizer and gloves on their institutions. However, there is lower availability of face masks (27.5%). Isolation gown (10.9%), eye protection (7.2%), N95 respirators (4.3%), and face shields (8%) than our study. This might be due to differences in the economic status of the country, which increases the capacity and distribution of protective equipment of the healthcare system, the difference in the study period. The study has certain limitations which must be acknowledged. First, the study was conducted only among HCPs working in public hospitals and didn´t represent HCPs working in different settings such as in private hospitals, health centers, and clinics. Second, the lack of standardized tools developed by WHO/CDC to assess HCP's level of preparedness, and finally this study cannot show cause and effect relationship since it is a cross-sectional type. Despite the identified limitations, these results contribute to the information relating to the overwhelming problem faced by HCPs especially related to the scarcity of PPE not only in Ethiopia but also at the global level

**Funding**: funding for this research project was gained from the Wolkite University.

## Conclusion

Based on our finding we can infer that the levels of health care providers´ preparedness and health care protection against the third wave of the COVID pandemic was found to be low. The finding also revealed that working experience, monthly income, and job description of health care providers were found to be statistically significant predictors of preparedness against COVID-19 pandemics. Federal Ministry of Health in collaboration with hospitals should pass emergency legislation to protect health care providers who are at high risk of exposure to COVID-19. This should include financial protections for healthcare workers who contract COVID-19 and supplement additional safety requirements for healthcare facilities. The private sector should work together to create a system to continuously track PPE supply chains and to facilitate rapid manufacture and equitable distribution of PPE at the very start of a new epidemic to prevent the critical shortages of PPE experienced in the COVID-19 pandemic. Moreover, hospitals should endeavor to establish and maintain crisis leadership skills in nurse professionals who can help foster and champion nurse preparedness and response at the highest levels of leadership.

### What is known about this topic

- *Healthcare providers are always at the forefront in the response to emerging infectious disease outbreaks; so, they are at higher risk of being infected with SARS-CoV-2 than the general population;*
- *The third wave of COVID-19 infection was first reported in the United Kingdom at the end of January 2020;*
- *Coronavirus cases have been rising in Africa since the start of the third wave in May 2021*.

### What this study adds

- *The levels of health care providers’ preparedness and health care protection against the third wave of the COVID-19 pandemic were found to be low;*
- *Findings indicate that government and other stakeholders should design interventions to increase health care workers' preparedness to respond to the ongoing pandemic and purchase an adequate supply of personal protective equipment to protect the health care workers*.
